# Single domain Camelid antibody fragments for molecular imaging and therapy of cancer

**DOI:** 10.3389/fonc.2023.1257175

**Published:** 2023-09-08

**Authors:** Shulin Li, Sanne Johanna Maria Hoefnagel, Kausilia Krishnawatie Krishnadath

**Affiliations:** ^1^ Center for Experimental and Molecular Medicine, Amsterdam University Medical Centers (UMC), University of Amsterdam, Amsterdam, Netherlands; ^2^ Cancer Center Amsterdam, Amsterdam, Netherlands; ^3^ Department of Clinical Geriatrics, Radboudumc, Nijmegen, Netherlands; ^4^ Department of Gastroenterology and Hepatology, Antwerp University Hospital, Antwerp, Belgium; ^5^ Laboratory of Experimental Medicine and Pediatrics, University of Antwerp, Antwerp, Belgium

**Keywords:** cancer, VHHs, mAbs, imaging, therapy

## Abstract

Despite innovations in cancer therapeutics, cancer remains associated with high mortality and is one of biggest health challenges worldwide. Therefore, developing precise cancer imaging and effective treatments is an unmet clinical need. A relatively novel type of therapeutics are heavy chain variable domain antibody fragments (VHHs) derived from llamas. Here, we explored the suitability of VHHs for cancer imaging and therapy through reviewing the existing literature. We searched the MEDLINE, EMBASE and Cochrane databases and identified 32 papers on molecular imaging and 41 papers on therapy that were suitable for comprehensive reviewing. We found that VHHs harbor a higher specificity and affinity compared to mAbs, which contributes to high-quality imaging and less side-effects on healthy cells. The employment of VHHs in cancer imaging showed remarkably shorter times between administration and imaging. Studies showed that ^18^F and ^99m^Tc are two optimal radionuclides for imaging with VHHs and that site-specific labelling is the optimal conjugation modality for VHHs with radionuclide or fluorescent molecules. We found different solutions for reducing kidney retention and immunogenicity of VHHs. VHHs as anticancer therapeutics have been tested in photodynamic therapy, targeted radionuclide therapy, immunotherapy and molecular targeted therapy. These studies showed that VHHs target unique antigen epitopes, which are distinct from the ones recognized by mAbs. This advantage means that VHHs may be more effective for targeted anticancer therapy and can be combined with mAbs. We found that high cellular internalization and specificity of VHHs contributes to the effectiveness and safety of VHHs as anticancer therapeutics. Two clinical trials have confirmed that VHHs are effective and safe for cancer imaging and therapy. Together, VHHs seem to harbor several advantages compared to mAbs and show potential for application in personalized treatment for cancer patients. VHH-based imaging and therapy are promising options for improving outcomes of cancer patients.

## 1 Introduction

Cancer is a major health challenge and one of the leading causes of death, accounting for a total of 10 million deaths in 2020 (1). Tobacco and alcohol usage, high body mass index, and unhealthy diet are common cancer risk factors (2, 3). Over the past decades, advances in cancer treatments include the implementation of multi-modality treatment strategies by a combination of (neo) adjuvant chemo (radio) therapy, surgery, targeted therapy and immunotherapy.

Early detection and adequate staging of cancer is pivotal for selecting adequate treatment. Imaging modalities on the anatomical level, which are often used for cancer detection and staging, include ultrasonography, x-ray and computed tomography (CT). For investigation of tumor dissemination, ^(18)^F-FDG positron emission tomography/CT (PET/CT) is a regularly used methodology. With PET/CT scanning, information regarding anatomy and metabolism is combined, which yields a higher sensitivity and specificity for tumor detection. A drawback of these imaging modalities, is that the spatial resolution is often insufficient to adequately characterize micro-metastases (4). Moreover, detection of specific molecules is not possible with the use of the currently applied radiological techniques. The development of molecular-oriented imaging modalities might further improve the specificity and sensitivity of imaging.

Beside challenges in cancer detection and staging, limitations of currently applied therapies include side effects and therapy resistance. Even in patients treated with curative intent, therapy resistance leading to disease recurrence is a major problem. With the current availability of high throughput molecular sequencing technologies for genomic and transcriptomic characterization, more personalized treatment strategies based on unique molecular characteristics of each patient are of great interest to improve therapy response. Personalized treatment regimens include targeted therapies. For example the first approved targeted agent tamoxifen, which binds to the estrogen receptor (ER), is now routinely used in breast cancer therapy (5). To assign patients to a particular targeted treatment regimen, it is of importance to investigate whether or not these targets are expressed in the cancer. Therefore, detection of target molecules is regularly performed in patient samples by histopathology using immunohistochemistry or fluorescence *in situ* hybridization. However, tumor heterogeneity for protein expression could lead to under- or overestimation of the expression of the target in the cancer. Moreover, acquiring patient material by surgery or biopsy is a burden for patients with potential complications.

Molecular imaging using nuclear or radiologic modalities with labeling of cell-surface receptors or antigens is a promising option to overcome limitations of current imaging modalities (6, 7). Ideally, molecular imaging would harbor multiple advantages such as non-invasiveness, reproducibility of results, easy access, more quantifiable and the possibility to examine whole organs within the human body. In the past, monoclonal antibodies (mAbs) have been considered the most specific probes for targeted imaging attributed to their binding specificity and affinity (8). Many mAbs have been investigated in clinical studies, however their size limits clinical implementation. For instance, Trastuzumab, which is an FDA-approved anti-HER2 mAb, has been used and investigated for imaging and treatment of patients with breast cancer overexpressing HER2 (9). The role of Trastuzumab in imaging was found to be limited because of its relatively poor tissue penetration and potential to miss small metastases. Furthermore, the impermeability of the blood brain barrier (BBB) for the molecule is a limitation, especially because breast cancers have a high propensity to metastasize to the central nervous system (10).

mAbs have limitations in application for cancer therapy. For instance, mAbs applied in antibody-drug conjugates (ADCs), which hold great promise for cancer treatment, but are also constrained by the complex structures of full-sized antibodies (11). Brentuximab Vedotin is an ADC approved plus Rituximab for management of patients with B-cell lymphoma, showed high levels of toxicity in nearly half of patients leading to discontinuation of the treatment (12). This is most likely because of a high off-target effect, with slow clearance from the circulation and long retention in non-targeted tissues. Moreover, the production of ADCs is complex because there is a limited range of conjugation methods, which at times results in heterogeneous mixtures. Accurate prediction of the ratio of drug over antibody is crucial to the pharmaceutical properties of ADCs, which requires sophisticated mass spectroscopic methods and time-consuming empirical optimizations (11).

To circumvent the disadvantages mentioned above, a variety of smaller biomarker-targeted proteins have been developed, which incorporated recombinant antibody fragments such as diabodies (50 kDa) and minibodies (80 kDa), and scFv-Fc fragments (105 kDa) (13). However, their utilization is limited through their inherent properties such as the fact that although they are relatively smaller than conventional mAbs (150 kDa) they are still relatively large for effective binding and have notable immunogenicity. Immunogenicity of drugs causes the generation of anti-drug antibodies, which is a problem for several approved mAbs such as Trastuzumab, Pertuzumab, Cetuximab, Foralumab and Rituximab (14).

VHHs seem to be superior biomarker-targeting proteins. A VHH is a single domain antibody fragment, which consists only of a heavy chain variable domain as can be derived from llamas. A VHH is the smallest naturally derived antigen-binding fragment (~15 kDa) with excellent biocompatibility. VHHs can be used for imaging and treatment of cancer similar to mAbs but with several advantages. Importantly, VHHs can infiltrate tumors more rapidly compared to mAbs due to their smaller size and aqueous solvability. Also VHHs are low-immunogenic in both mice and humans due to the absence of a Fc region. The superior specificity of VHHs results from their capability of binding to specific epitopes that cannot be reached by conventional mAbs. Studies have shown that VHHs allow higher tumor-to-background (T/B) ratios than conventional antibodies in molecular imaging in vivo. This is attributed to the highly specific binding and accumulation of VHHs in tumor and rapid clearance of unbound constructs from the body leading to less background signal (8). Moreover, this leads to a lower toxicity of VHHs compared to conventional antibodies. One report suggested that VHH-drug conjugates are desirable alternative for conventional ADCs (11). In addition, VHHs are stable and can be chemically modified (15). Production of VHHs linked to cytotoxic drugs is better feasible than the production of ADCs because VHHs can easily be produced in mammalian cells, bacteria and yeast (16–18).

In this review, we explored the suitability of VHHs-based imaging and therapy through reviewing previous preclinical and clinical studies.

## 2 Methods

To explore the suitability of VHH-based imaging and therapy of cancer, a literature review search was conducted in MEDLINE, EMBASE and Cochrane Library database. The combination of search terms “cancer*”, “neoplas*”, “tumo*”, “malignan*”, “metasta*”, “carcino*”, “adenocarcino*”, “adeno-carcino*”, “onco*”, “neoplasms”, “oncology” or “oncogenes” with “single domain antibodies”, “antibod*” “anti-bod*”, “nanobod*” “nano-bod*” “llama*” “lama*” “VHH” or “VHHs” was used to retrieve all papers from the Medical Library from Amsterdam University Medical Centers (Location University of Amsterdam) focused on VHHs in cancer until April 19th, 2022. The detailed search was performed as displayed below. Firstly, abstract screening was performed for the eligibility by reading the title and abstract of the paper. Review papers, conference abstracts, papers without full text, papers not written in English, and papers that did not focus on VHHs in cancer research were excluded. After abstract screening, data extraction was performed and the most important findings were displayed in the tables.

## 3 Results

**Figure 1:**
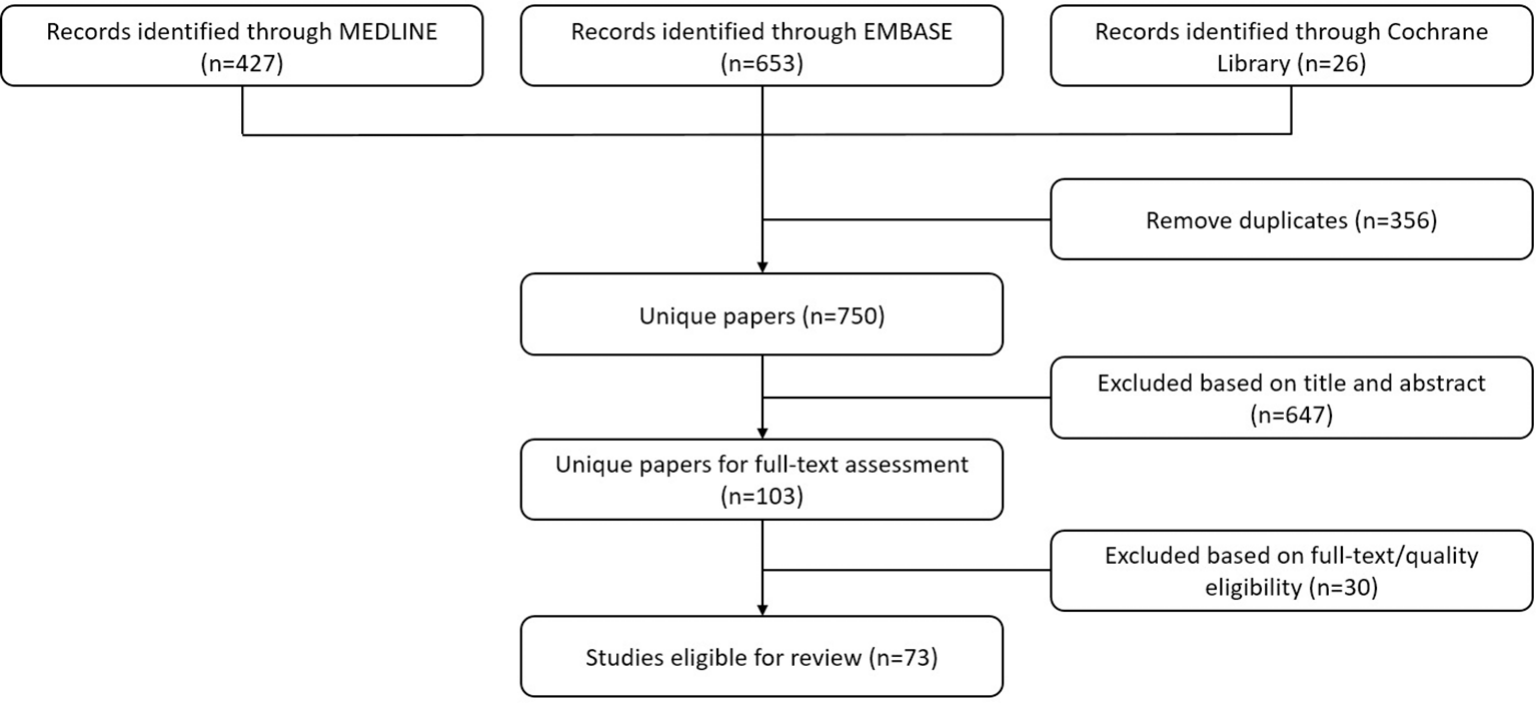
Flowchart displaying the processing of the literature. The authors performed screening for all retrieved papers and removed duplicates in compliance with the criteria set based on the theme of this literature review.

A total of 1106 papers were retrieved. After removing duplicates, 750 eligible papers were identified and were thoroughly screened for inclusion in this review. There were 73 papers that were eligible for inclusion (**Figure 1**), including 71 papers published until April 19th, 2022 and 2 papers published later than April 19th, 2022 from our research group. Upon reviewing we were able to include 32 papers for molecular imaging of cancer (**Supplementary Table 1**) and 41 papers for therapy of cancer (**Supplementary Table 2**).

### 3.1 Molecular imaging of cancer with VHHs

There were 32 studies included that investigated VHHs for their ability as molecular imaging agents. The general characteristics of the studies including types of imaging, types of experimental models and types of cancer are displayed in **Supplementary Table 1**. 20 studies focus on radiolabelled imaging and 12 studies on optical imaging. 13 studies investigated VHHs in in vitro and in vivo models, 9 studies investigated VHHs in in vitro, in vivo and ex vivo models, 7 studies investigated VHHs only in in vivo models, 1 study investigated VHHs in in vitro and ex vivo models, 1 study investigated VHHs in in vivo and ex vivo models, only 1 study investigated VHHs in a clinical phase I trial. The types of cancers that were investigated included breast cancer (n=8), lung cancer (n=4), ovarian cancer (n=4), glioblastoma multiforme (n=3) colorectal cancer (n=2) melanoma (n=2), lymphoma (n= 2), head and neck cancer (n=1), prostate cancer (n=1), multiple myeloma (n=1), epidermoid carcinoma (n=1), human epithelial cancers (n=1) and EGFR signaling abnormal cancers (n=2).

#### 3.1.1 Radiolabeled imaging with VHHs

Among the 21 studies on radiolabelled imaging, the radionuclide-based VHHs were directed against tumor targets and proteins important in immune response including: epidermal growth factor receptor (EGFR) (19, 20), macrophage mannose receptor (MMR) (21, 22), human epidermal growth factor receptor type 2 (HER2) (23–30), carcinoembryonic antigen (CEA) (31), signal regulatory protein alpha (SIRPα) (32), Class II major histocompatibility complex antigens (MHC-II) (33), CD8 (34, 35), carbonic anhydrase IX (CAIX) (36), spliced EIIIB (EDB) domain of fibronectin (FN) (37), multiple myeloma M-protein (38) and prostate-specific membrane antigen (PSMA) (39).

VHHs conjugated with radionuclides have been successfully exploited in research settings with the single-positron emission tomography (SPECT) combined with micro-computed tomography (micro-CT) for imaging in cancer (19, 22, 25, 26, 31, 32, 36, 38, 39). In these studies, ^99m^Tc, ^111^In, ^225^Ac and ^131^I, ^177^Lu are radionuclides that were used for imaging. These studies show that radiolabelled VHHs exerts efficient tumor targeting, low non-target binding and excellent imaging results. VHHs show high specificity to detect primary tumors and metastatic sites in multiple models of melanoma, breast cancer, ovarian cancer, early pancreatic lesions such as pancreatic intraepithelial neoplasia and advanced pancreatic ductal adenocarcinoma with excellent clarity and signal-to-noise ratios through PET/CT imaging (30, 37). One study showed that humanized anti-CEA ^99m^Tc-VHHs display high targeting specificity with low signals in non-targeted organs, high heat-stability and rapid renal clearance using SPECT/micro-CT in a colon carcinoma mice model (31). High tissue penetration and high tumor targeting allow VHHs to easily reach brain targets by overcoming the BBB. For example, ^99m^Tc-labeled VHHs could successfully target SIRPα in glioblastoma (32). Puttemans et al. also found that VHHs labelled with either [^131^I] or [^225^Ac] display a high and specific tumor uptake in HER2 positive brain metastasis lesions, whereas their counterparts mAbs Trastuzumab-[^131^I] and Trastuzumab-[^225^Ac] are unable to accumulate in intracranial tumors (26). The study showed that ^111^In-labeled anti-HER2 VHHs display high specific uptake in HER2 positive brain tumor from 1 h up to three days post injection, whereas ^111^In-labeled mAb Trastuzumab causes high non-specific uptake in highly vascularized organs including heart, spleen and liver (26).

VHHs can also be used for detection of subset of immune cells. For instance, ^99m^Tc-labeled anti-MMR VHHs accumulate in hypoxic regions by targeting a specific subset of tumor-associated macrophages (TAMs), which are associated with strong angiogenic properties. This has been visualized through SPECT/micro-CT imaging (22). There are also multiple examples of the use of VHHs to detect biomarkers for response to immunotherapy. Real-time PET imaging of ^89^Zr-labeled anti-CD8^+^ and CD11b^+^ VHHs show the potential to assess the dynamic distribution of CD8^+^ and CD11b^+^ cells as predictive biomarkers for response to anti-PD-1 treatment (35). Furthermore, assessing the distribution of intratumoral CD8^+^ T cells by PET imaging after application of ^89^Zr-labeled anti-CD8^+^ VHHs is associated with the response to CTLA-4 (cytotoxic T-lymphocyte-associated protein 4) therapy (34). One study demonstrated that ^99m^Tc-VHHs and SPECT/micro-CT successfully real-time monitor the expression change of M-protein in a mice model with multiple myeloma, with high uptake in M-protein expressing malignant plasma cells in the blood and no uptake in healthy mice (38). ^18^F-labelled anti-MHC-II VHHs successfully detected inflammation through PET/CT imaging at melanoma tumor growth sites before tumors were visible or detectable by palpation (33). ^18^F-labelled anti-MHC-II VHHs and anti-CD11b VHHs, which bind to targets expressed by multiple immune cells, detected primary melanoma, as an alternative to melanoma-specific markers in a melanoma mice model (33).

As a result of their small size, VHHs are able to rapidly shift from blood to targeted tissues and blood clearance and elimination is also relatively fast. This makes the time between administration and imaging relatively short, allowing a high image quality at early time points following injection. ^18^F-labelled anti-MHC-II VHHs were rapidly cleared from circulation 20 min post injection in a melanoma mice model (33). In another study, tumor imaging was possible as early as 3 h following the administration of radiolabeled anti-EGFR VHHs (20), compared to 16 h for radiolabeled anti-EGFR mAbs. Moreover, imaging by the latter was accompanied with relatively high background signals from the liver and the gastrointestinal tract (40). One study reported that fast blood clearance limits the absolute uptake of the VHH and thereby limits imaging of low-abundant targets in therapeutic applications (36). To prolong the blood residence time of VHHs and increase tumor uptake, one feasible option is the introduction of an albumin-binding domain to the VHHs while preserving the inherent high affinity and specificity of the molecules (36).

In a comparative study of in vivo tumor uptake of VHHs- and mAbs-coated nanoparticles in CT molecular imaging, VHHs with longer blood residence time (20 h for VHH- and 11 h for mAbs-coated nanoparticles) failed to make up for its lower binding affinity, leading to significantly lower in vivo tumor accumulation of VHHs than mAbs (41). This data suggests that VHHs with a long circulation time but low target affinity perform less with regards to tumor accumulation and imaging. The binding affinity can have more impact on tumor accumulation than blood residence time in certain conditions, but studies to confirm this observation are warranted.

##### 3.1.1.1 Reduction of renal retention and liver uptake of radiolabelled VHHs

Relative high kidney retention is the major limitation of radiolabelled VHHs for molecular imaging (42, 43). High kidney uptake is a problem for clinical translation for treatment and imaging of kidney diseases, because it may interfere with the detection for primary or metastatic tumor lesions. For instance, it could impact the visualization of minor tumor lesions in the proximity of the kidneys and especially with the staging of prostate cancer. The kidney retention is a universal phenomenon that exists in all VHHs labeled with radiometals (for instance, ^99m^Tc, ^111^In, ^225^Ac) or radiohalgen (for instance, ^131^I, ^18^F). Among the 20 studies with radiolabelled imaging, 13 studies show high uptake of VHHs in the kidneys (19, 20, 22, 23, 25–28, 30–32, 34, 38).

The number of polar residues in C-terminal amino acid tag is the predominant reason for kidney retention of VHHs (44). One study has shown that kidney retention of radiolabeled VHHs can be significantly reduced though the use of an untagged C-terminus in combination with the plasma expander gelofusine (45) or the introduction of brush border enzyme-cleavable linkers in the prosthetic moiety because the brush border enzymes can impede reabsorption of radiometabolites (46). Another study showed that VHHs lacking the C-terminal His tag reduce 60% of kidney uptake compared to regular VHHs (30). Untagged VHHs showed reduction of 70% in kidney accumulation compared to Myc-His-tagged VHHs, whereas co-infusion of untagged VHHs with the plasma expander gelofusine achieved a 90% drop in kidney accumulation (45). In addition, the study on ^111^In- anti-PMSA-VHH-c-myc-his, Chatalic and colleagues demonstrated that the his-tag is responsible for the high retention of radiolabeled VHHs in the kidneys (39) and D’Huyvetter also confirmed this conclusion (45). ^111^In- anti-PMSA-VHH-cys formed by cysteine replacing c-myc-His shows a reduction of renal uptake without reduction of tumor targeting at 4 h after injection (39). ^111^In-anti-PMSA-VHH-cys in combination with gelofusine and lysine co-administration showed a further reduction in renal uptake (39). Moreover, one study showed that anti-HER2 VHH, ^99m^Tc-7C12, co-injected with gelofusine and lysine reduce 45% of renal retention, and increase the tumor uptake (47). Similarly, other studies about ^177^Lu-labeled anti-HER2 VHHs demonstrated that the kidney retention can be dramatically reduced by the removal of polar residues, and is further decreased by co-infusion of gelofusine (drop of 95%) (42).

The renal retention of VHHs labeled with radioiodine using Iodogen is relatively low as an exception, which is probably due to their rapid dehalogenation in vivo causing free VHH and Iodogen (48). Another report suggests that renal toxicity of radiolabeled protein results from the long residence time of radiometabolites after lysosomal proteolysis, glomerular filtration and reabsorption into renal cells (49). Therefore, additional studies should be performed to determine whether radioactivity retention in kidneys can result from free radioiodine generated by lysosomal proteolysis, to study further strategies to decrease kidney retention.

In addition to the high kidney uptake, high liver uptake is also of concern for the translation of radiolabelled VHHs to the clinic. Among 20 studies, 7 studies highlighted the high liver uptake (20–23, 26, 28, 32). Although high liver uptake might interfere with the detection for primary or metastatic tumor lesions, toxicity is not as much of a concern as radiation dosimetry results proved that the high liver uptake was not related to any hematological or biochemical abnormalities with clinical significance in human subjects (50). The high liver uptake is probably ascribed to high expression of the target antigen in the liver. For instance, the liver was highly radioactive after treatment with [^177^Lu]Bz-DTPA-EGF for binding to EGFR for glioblastoma in mice (51). The high radioactivity could be significantly reduced by injection of unlabeled EGF before treatment, without interfering with radioactivity of the tumor target (51). This phenomenon was also observed in another study in mice (52). Furthermore, the overall charge and lipophilicity of VHHs also contributes to the high radioactivity in liver (20), which can be overcome by conjugating a certain prosthetic group to VHHs to increase hydrophilicity.

##### 3.1.1.2 Suitability of multiple radionuclides and modalities of conjugation

Various factors should be taken into account when labelling VHHs. It is important to note that the targeting property and pharmacokinetics of the protein could be altered by protein labelling, particularly for small proteins like VHHs. Synthesis of radiolabelled VHHs by conjugating VHHs to radionuclides is an important factor, which determines the characteristics of the conjugated VHHs. There are nine kinds of radionuclides used for radiolabeled imaging, including: ^18^F, ^111^In, ^99m^Tc, ^64^Cu, ^89^Zr, ^68^Ga, ^225^Ac, ^131^I and ^177^Lu. Here we discuss the suitability of radionuclides for imaging.

^125^I is a widely studied radiopharmaceutical that is transported into cells and incorporated directly into DNA during the S phase in cell division (53). However, ^125^I has limitations when used as a radiotherapeutic agent because of its relative lack of specificity for tumor cells, ability to target cells only in S phase, its extensive deiodination in the liver and high radiotoxicity to off target mammalian cells (53). Moreover, limited stability and early release of labelled iodine from their conjugated VHH before reaching their target protein might limit clinical usefulness, as was shown for the application of ^125^I-labelled EGF in an in vivo model of an EGFR overexpressing tumor (54).

Another radionuclide is ^111^In-oxine, which is a lipophilic chelate that is used for radiolabeling for targeted radiotherapy. ^111^In-oxine is only used for radiolabeling and is not suitable as a radiotherapeutic agent because it internalizes non-specifically into both healthy and cancer cells. Several studies investigated the possibility to make ^111^In-oxine more specific for cancer cells, to increase its potential as a radiotherapeutic agent in cancer (53). Next to the lack of discrimination between healthy and cancer cells, it has been reported that ^111^In-labelled EGF cannot discriminate between high and moderate EGFR overexpression (20).

^177^Lu is a superior radionuclide candidate for treatment of small tumor cell clusters, since it emits relatively low-energy beta particles, reducing radiotoxicity for adjacent healthy cells (51). The limitation of ^177^Lu is on its safety. It has been reported that patients with neuroendocrine tumors develop myelodysplastic syndrome and acute myeloid leukemia after treatment with targeted radionuclide therapy with ^177^Lu peptide (55).

^68^Ga is another commonly used radionuclide and is conjugated to an antibody for molecular imaging. ^68^Ga-labeled VHHs applied in immunoPET scanning show good feasibility for the evaluation of HER2 status in patients with breast cancer metastases in a phase I clinical study (23). However, the short half-life of ^68^Ga limits flexibility of imaging timing with for example a maximum imaging time of 90 min after administration of ^68^Ga-HER2-VHHs when applied in patients with breast cancer (23).

^18^F could be a more promising radionuclide for labeling VHHs because ^18^F has a more than threefold lower energy and tissue range compared to ^68^Ga (27), resulting in improved spatial resolution and avoiding side effects of the radionuclide in the surrounding healthy tissues. The energy of the emitted radionuclide is negatively correlated to the resolution of the imaging. ^18^F-labelled anti-HER2 VHHs showed a tumor-to-background and tumor-to-muscle ratio of 13 and 34, respectively, compared to 3 and 10 for ^18^F-labelled anti-HER2 affibodies, and 2 and 7 for ^18^F-labelled anti-HER2 diabodies (29). This suggests that the combination with VHH enhances the suitability of ^18^F used for imaging. Moreover, ^18^F-labeled compounds have superior properties with respect to electric charge and metabolization. Blykers et al. show significant lower kidney, liver and spleen uptake of ^18^F-anti-MMR VHH compared to ^99m^Tc-anti-MMR VHH in MMR-deficient tumor models and low bone uptake indicated that no in vivo defluorination occurred (21). The fluorinated tracer showed decreased binding to extratumoral sites, while preserving tumor targeting (21). Other studies confirmed low kidney retention with ^18^F-labeled VHHs in comparison with ^68^Ga-, ^99m^Tc-, ^177^Lu- and ^111^In-labeled VHHs (28, 56). Additionally, ^18^F-labelled anti-HER2 VHHs are not retained in the liver, a frequent site of metastases for HER2-positive breast cancers, which offers a potential advantage compared to other HER2-specific immunoPET agents such as ^89^Zr-DFO-Trastuzumab, which exhibits significant accumulation in the liver (57). Moreover, ^18^F-labeled VHHs show high stability in vitro and in vivo, without presence of free ^18^F, [^18^F]-SFB or aggregates (29). [^18^F]-SFB is used as a prosthetic group for fluorination of peptides and proteins. In addition, ^18^F has a longer half-life providing the flexibility on time points of imaging in case imaging needs to be delayed due to problematic background activity.

^99m^Tc is preferred for labeling radiopharmaceuticals due to its low cost and its favorable physical characteristics, such as suitable half-life and lower gamma emission energy (58). Scientists have attempted to replace ^131^I-, ^123^I-, ^111^In-, ^67^Ga-labeled compounds with corresponding ^99m^Tc-labeled compounds. Ethylenedicysteine has been successfully used for easy and efficient labelling ^99m^Tc to certain compounds with high radiochemical purity (59).

Overall, ^18^F and ^99m^Tc are two optimal radionuclides for imaging. Four of the 20 radiolabeling imaging studies used ^18^F (21, 27, 29, 33) and seven studies used ^99m^Tc (19, 20, 22, 28, 31, 32, 38).

The conjugation of antibodies is commonly performed by direct radiolabelling or by using a bifunctional chelating agent (60). Bifunctional chelating agents are used to stably link the radiometal to the carrier of the radiopharmaceuticals, for instance, VHHs (61). To protect healthy cells and tissues from radiation damage, it is imperative to utilize stable and inert complexes of radionuclide-antibody conjugates for imaging. Above all, an ideal labelling modality should preserve intrinsically high specificity and affinity of VHHs and ensure homogeneity of the generated VHH tracers resulting in reproducible pharmacokinetic and pharmacodynamic properties. The site-specific labeling of VHHs with a radionuclide via a thio-ether bond by introduction of unpaired cysteine at the carboxyl-terminal end of the VHH was demonstrated to be an effective radiolabelling modality with a clinical translational value, because the labelled VHHs retained their inherent superior characteristics and homogeneity (25). There are six imaging studies in this review employing site-specific labelling modalities for forming VHH conjugates (25, 33, 39, 62–64).

##### 3.1.1.3 The application of VHHs in MRI

In addition to radiolabeled imaging, VHHs also can be conjugated with magnetoliposome (MLs) to serve as contrast agents for magnetic resonance imaging (MRI) (24).These conjugates can serve as imaging probes for locating and diagnosing cancerous lesions (65). One study developed an anti-HER2 VHH-based targeted ML for achieving intelligent MRIs of breast cancers (24). Properties of specificity, internalization and binding capability of anti-HER2 VHHs were retained after conjugation of the anti-HER2 VHHs with the MLs. MRI imaging of HER2 positive cells with anti-HER2 VHH-MLs showed high specificity with enough contrast to HER2 negative cells even at low cell density, which is attributed to more potent internalization of anti-HER2 VHH-MLs compared to mAbs Herceptin-MLs.

#### 3.1.2 Optical imaging

**Figure 2:**
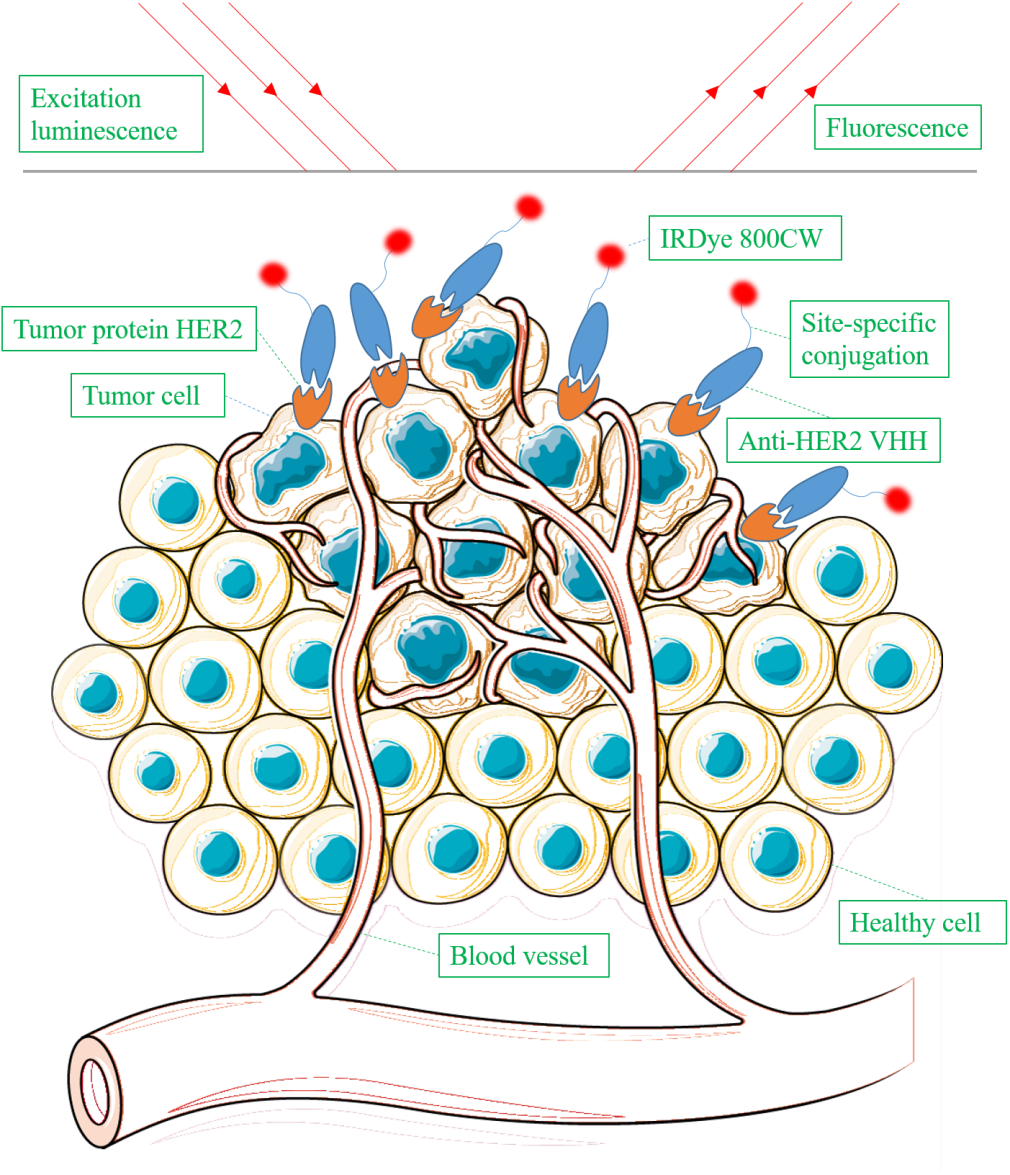
Schematic overview of detecting tumor protein HER2 using IRDye 800CW-labeled anti-HER2 VHH via optical imaging.

There are 11 studies focusing on the application of VHHs in optical imaging (8, 11, 41, 62–64, 66–70). Optical imaging uses light to obtain detailed images of molecules (**Figure 2**). As observed in the study of molecular optical imaging with IRDye 800CW (IR)-labeled anti-HER2 VHHs, VHHs-based optical imaging exhibits two attractive characteristics: rapid accumulation in the tumor and high T/B ratio. T/B ratio is determined by two factors: specific target binding capability and rapid clearance of unbound antibodies in the body. VHHs generally exhibit higher T/B ratios than conventional mAbs when used for molecular imaging in vivo. VHHs have better tissue penetration and fast clearance from the circulation, whereas mAbs display slow clearance. These pharmacokinetics are of importance for optimizing dose and time points of administration and imaging in clinical practice.

Specific target binding capability has been investigated in multiple studies. VHHs-IR were highly specific with accumulation only in HER2-positive tumors, as opposed to the mAb Trastuzumab-IR, which accumulated in both HER2-positive and HER2-negative tumors (62). In a glioblastoma multiforme (GBM) mice model and in ex vivo human GBM tissue, IRDyeCy5.5-labelled anti-IGFBP7 VHHs did bind to GBM vessels at 10 min to 24 h after injection, but did not to normal brain vessels (67). Alexa Fluor 647 (a NIR dye)-labelled anti-MHC-II VHHs exert high specificity, high cytotoxicity and fast cellular internalization (within 1 h) in localized and metastatic tumor cells in B-cell lymphoma models, with signals from 30 min to 96 h after injection (11). Imaging of anti-EGFR VHHs showed highest fluorescence at 2 h post injection of anti-EGFR VHHs, compared to 24 h for anti-EGFR mAbs Cetuximab (70).

Moreover, excellent T/B ratio and good contrast between tumor and background enable surgical resection guided by fluorescence derived probes specifically accumulated in the tumor. NIRF-dye AF680-labelled VHHs displayed a 6-fold faster tumor accumulation and 2-fold better T/B ratio than mAbs at the same dosage in a lymphoma mice model (8). One study showed that the anti-HER2 VHHs-IR significantly outperform Trastuzumab-IR on overall T/B ratio and serves as a useful imaging-guide in a HER2-positive xenograft (62). Anti-HER2 VHHs-IR have a ∼20 times faster tumor accumulation than the anti-HER2 mAbs Trastuzumab-IR (62). This was associated with intravenous administration of VHHs-IR a few hours before surgery, compared to 3 days for the mAb Trastuzumab-IR. There is evidence that anti-CAIX VHHs-IR can provide pre-, intra-, and postoperative optical imaging of (pre-invasive) breast cancer in a xenograft breast cancer mouse model (64).

Expression of tumor markers may alter in the primary tumor and metastatic lesions during cancer progression. Employing one unique probe might be insufficient to adequately image all tumor lesions. This was addressed in a recent study, which investigated whether a combination of two optical VHH probes, which specifically recognize two independent breast cancer markers, could improve tumor imaging by increasing the T/B ratios. As expected, application of two optical VHH probes effectively increased the T/B ratio compared to that of a single VHH probe, with successful detection of small metastases (69). In this way, the expression status of different tumor markers can be observed simultaneously within the same tumor, enabling a more complete tumor characterization. Another benefit is that it provides a rapid, non-invasive assessment of tumor marker expression in a pathological setting. Moreover, combining multiple optically labelled VHHs to target neighboring molecules can be performed without steric hindrance compared to mAbs. Imaging by using the combination of different probes has also been reported in other studies (71–73).

Moreover, bimodal VHHs can be developed to enable application of one agent for multiple imaging and/or treatment modalities. In addition to conventional optical imaging, a bimodal VHH that constituted by site-specific dual-labeling of ^111^In and IRDye700DX was investigated and developed for SPECT imaging and targeted photodynamic therapy (63). The bimodal ^111^In-labeled DTPA-IRDye700DX VHH was found to specifically accumulate at the EGFR overexpressed tumor sites in mice bearing A431 xenografts. This enabled successful visualization with both near infrared fluorescence and SPECT imaging. This novel bimodal VHH displayed a high internalization ratio and retained high specificity and affinity. Furthermore, application of a bimodal VHH can circumvent the limitations of a single imaging modality. This bimodal VHH could potentially be applied in oncologic surgery for EGFR overexpressing tumors, such as lung cancer, head-and-neck cancer, brain tumors and bladder cancer (63).

Another study underscored the potential of VHHs for monitoring the efficacy of mAb Daratumumab therapy, since they can still detect their target CD38 after binding of Daratumumab (66). This is because anti-CD38 VHHs bind to different epitopes with the ones that Daratumumab does. Therefore, VHHs hold promise as novel tools, which can be applied collaboratively with Daratumumab for diagnosis and treatment of CD38-expressing malignancies.

A limitation of most of the imaging studies was neglecting dose optimization (62, 70). The excess antibodies may have contributed to non-specific accumulation in non-target organs (74). Similar as for the application of VHHs in radiolabeled imaging, the VHHs’ short circulation half-life might limit clinical application in optical imaging. Efforts to prolong the half-life of VHH in circulation is performed by engineering strategies, such as pegylation, multimerization and fusion (75). In a comparative study, the synthesis and pharmacokinetic properties of bivalent VHH EG2-hFc (80 kDa) and pentavalent V2C-EG2 (128 kDa) constructs based on monovalent EG2 were described (68). As expected, the half-life of EG2-hFc and V2C-EG2 in circulation was improved and EG2-hFc showed a significant increase in affinity for its target antigen compared to the monovalent VHH. The EG2-hFc construct effectively targeted intracranial brain tumors in in vivo molecular imaging with a good balance between affinity, serum half-life, molecular size and tumor penetration. Such a balance with effective in vivo molecular imaging and therapy was achieved only for the bivalent VHH constructs but not in higher-valent constructs (68),. In this way, excessive repeat dosing can be avoided, which reduces the burden for patients during molecular imaging.

Conjugation of a fluorophore to a VHH for optical imaging might affect its binding affinity. To resolve this problem, protein site-directed conjugation has been developed to replace conventional random conjugation and to avoid affinity loss (62). Conjugating fluorophores specifically to the C-terminal region of a VHH is a good option, because the C-terminus is situated opposite to its epitope-binding region. The VHHs applied for recognition of two independent breast cancer markers as described above, were site-specifically conjugated to their respective fluorophores using a cysteine that was introduced in the C-terminal region (69), avoiding the effect of fluorophore conjugation on the binding affinity.

##### 3.1.2.1 Reduction of immunogenicity of VHHs

Another major limitation of the studies is that side effects due to immunogenicity of the VHHs has not been well reported. The immunogenicity of VHHs is still an issue when it comes to its clinical applications although it is lower than the immunogenicity of full size antibodies (76). The anti-VHHs immune response could be elicited in humans due to the non-human origin of the VHHs, impeding the capability of target binding of VHHs and causing allergy-like symptoms when repeatedly administrated (77). The VHH as the smallest antigen-binding fragment contains no Fc fragment, which is an advantage compared to full size mAbs. The Fc fragment of antibodies can bind to cellular receptors from phagocytic cells leading to phagocytosis. Immune recognition activated by the Fc domain also can lead to rapid clearance of antibody-coated nanoparticles from the bloodstream, leading to the reduction of tumor uptake (78). Given that the immunogenicity of VHHs might compromise their usefulness, humanizing VHHs by drafting the complementarity determining regions onto a human framework and keeping the original affinity has been undertaken previously (50). The humanization of VHHs generates its corresponding non-immunogenic derivatives, and can maintain the complete antigen-binding properties of the original VHHs. Retaining high affinity, specificity and heat-stability as well as effective conjugation with ^99m^Tc has been observed in humanized graft VHHs compared to original VHHs (31). Therefore, grafting the antigen-binding loops of a broad variety of VHHs onto a universal, humanized scaffold seems an effective strategy to remove immunogenicity (31).

### 3.2 Therapies of cancer with VHHs

We reviewed 41 studies that investigated VHHs for their ability as anticancer therapeutic agents. The general characteristics of the studies including types of therapy, types of experimental model and types of cancer have displayed in **Supplementary Table 2**. 24 studies investigated VHHs in both of in vitro and in vivo models, 8 studies investigated VHHs only in in vitro models, 4 studies investigated VHHs in in vitro, in vivo and ex vivo models, 2 studies investigated VHHs in in vitro and ex vivo models, 1 study investigated VHHs in in vivo and ex vivo models, 1 study investigated VHHs in an in vivo model, 1 study investigated VHHs in in vitro, in vivo and in patients. 26 studies focused on molecular targeted therapy, 5 studies on targeted radionuclide therapy, 5 studies on photodynamic therapy and 5 studies on immunotherapy. The types of cancers that were investigated included breast cancer (n=7), ovarian cancer (n=4), multiple myeloma (n=4), pancreatic cancer (n=4), leukemia (n=4), colorectal cancer (n=2) melanoma (n=2), head and neck cancer (n=2), gastric cancer (n=2), cervical cancer (n=2), esophageal adenocarcinoma (n=1), lung cancer (n=1), epidermoid carcinoma (n=1), hepatocellular carcinomas (n=1), Met-overexpressed cancer (n=1), EGFR-overexpressed cancer (n=1), esophageal adenocarcinoma premalignant lesion (n=1) and HPV induced cancer (n=1).

#### 3.2.1 Photodynamic therapy

There are five studies on photodynamic therapy in this review (79–83). Photodynamic therapy involves a light source of a particular wavelength, a photosensitizer (PS) and oxygen to destroy cancer cells (84). Most PSs used in the clinic are hydrophobic, which makes them easier to bind to cells, but also leads to non-specific binding. In several studies hydrophilic PSs were created by conjugating the PS to a VHH. The VHH-PS conjugates proved to be able to more specifically target antigens on tumor cells (79, 80, 82, 83).

Four research groups have confirmed the efficacy of VHH-PS in preclinical studies (79, 80, 82, 83). All studies used IRDye700DX as PS to form VHH-PS conjugates with VHHs. For example, anti-Met VHH-PS specifically killed targeted tumor cells upon illumination. The binding affinity of the target cells was very high (79). Another study showed that a conjugate of anti-EGFR VHH and hydrophilic PS IRDye700DX strongly binds to high EGFR expressing tumor cells and hardly bind to low EGFR expressing tumor cells, whereas PS IRDye700DX alone doesn’t bind to any cells (82). Anti-EGFR VHH-PS monovalent conjugates resulted in tumor necrosis in approximately 90% of tumor cells, which was significantly better than the mAbs Cetuximab-PS in an orthotropic mouse tumor model of head and neck cancer (82). Notably, the anti-EGFR VHH-PS conjugates were observed to homogenously distribute throughout the solid tumor, which is beneficial for the treatment efficacy. Another study confirmed that anti-EGFR VHHs are more homogeneously distributed throughout the tumor, whereas the anti-EGFR mAbs Cetuximab are confined to the core of the tumor (70).

Rapid penetration of VHHs was also displayed in the application of PDT. Effective illumination could already be performed 1 h post administration of VHH-PS conjugates, whereas illumination effects could only be seen 24 h post-administration with mAbs Cetuximab-PS conjugates (82). Nevertheless, several strategies to enhance the efficacy of VHH-PS were investigated. Cell-penetrating peptides (CPPs) are short peptides which facilitate CPP/drug complexes to translocate across the cell membrane (85). CPP increased the internalization of VHH-PS conjugates but slightly decreased their affinity, leading to reduced PDT efficacy (83). Biparatopic antibodies bind to two distinct epitopes of the same antigen (86). The potential of biparatopic antibodies in PDT has been explored. The biparatopic VHH-PS conjugates exert more toxic effect to tumor cells because they could deliver more PSs and enhance internalization (82). One study reported that internalized biparatopic VHH-PS significantly increased phototoxicity compared to monovalent VHH-PS conjugates located on the outer of cells (80). The mixture of Met targeted VHH-PS and EGFR targeted VHH-PS could further enhance therapeutic efficacy of VHH-PS and decrease potential therapy resistance (79). In addition, the degree of conjugation of the VHH-PS impacts treatment efficacy and photocytoxicity (82). Approximately only 50% of monovalent VHHs form VHH-PS with PSs, whereas for an equal quantity of biparatopic VHHs, all VHHs were conjugated with PSs. The competition between conjugated monovalent VHH-PS and unconjugated VHHs for target antigen EGFR leads to lower phototoxicity and T/B ratio compared to biparatopic VHHs (82).

It is speculated that although the advantage of PDT lies in restricting light dose specifically to tumor cells, normal cells near the tumor to some extent may encounter illumination. To eliminate unwanted effects on normal cells near the tumor from low T/B ratio, it is important to set an appropriate threshold dose for PDT. Heukers and colleagues performed co-culturing of high- and low- EGFR expressing tumor cells and adjusted the illumination and found that VHH-PS could be 100% specific to high EGFR expressing cells and safe for low EGFR expressing cells (80), which is in agreement with other studies (87, 88).

Moreover, as an alternative to PSs, branched gold nanoparticles can be used to form VHHs-branched gold nanoparticles conjugates. These have been applied for PDT and showed effective therapeutic efficacy for cancer treatment in a SKOV3 (HER2^+^) cell model (81).

#### 3.2.2 VHH-based immunotherapy

**Figure 3:**
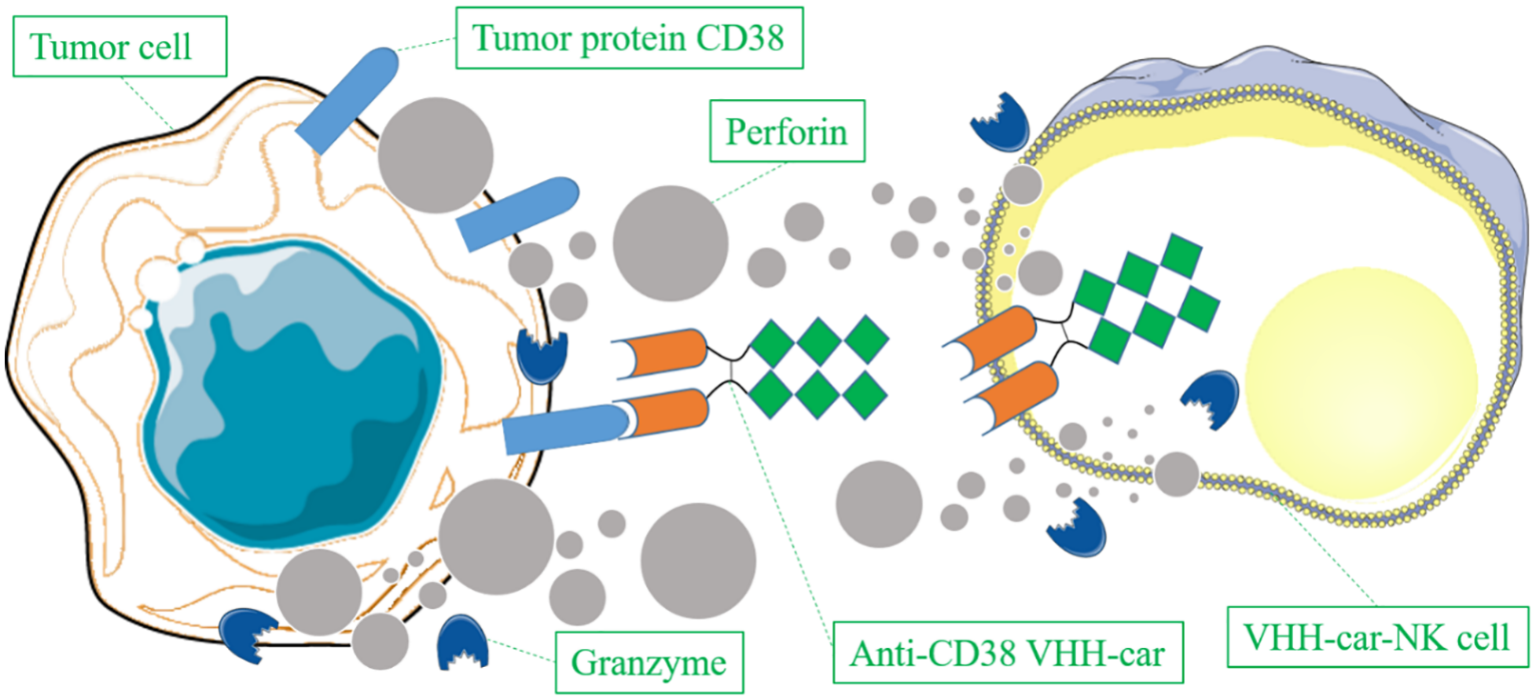
Schematic representation of VHH-car-NK cells specifically binding to CD38-positive tumor cells through expressing anti-CD38 VHH-cars and lysing the tumor cells via secreting perforin and granzyme.

CAR T-cell therapy is a special type of immunotherapy in which T-cells isolated from patients are genetically modified to express specific receptors directed against antigens of cancer cells, leading to T-cell attacks to destroy cancer cells (89). We found five studies on VHH-based immunotherapy (90–94), among which four studies on VHH based CAR T-cell therapy (91–94). Optimization of CAR T therapy has been investigated through various approaches. One study transduced the human natural killer cell line NK-92 to stably express VHH-based chimeric antigen receptors (VHH-CARs) specific for CD38 and as such developed anti-CD38 VHH-CAR NK-92 cells which exhibited promising efficacy for killing multiple myeloma cells in primary human bone marrow samples (91) (**Figure 3**). NK-92 cells are considered as a ready-to-use reagent compared to regular CAR T therapy which applies T cells that need to be isolated from patients. Phase-I clinical trials have confirmed that NK-92 cells (without VHH-CARs) have a high safety profile when used for patients (95, 96). Unfortunately, there are no clinical trials on anti-CD38 VHH-CAR NK-92 yet.

B-cell maturation antigen (BCMA) of multiple myeloma is one of most studied tumor antigens among CAR T therapy, which showed promising efficacy and clinical manageable side effects in different clinical trials on multiple myeloma (97–99). Single VHH targeting one epitope of BCMA in CAR-T therapy has also been explored (92). Anti-BCMA CAR-T cells produced by using a humanized VHH sequence were able to kill lymphoblastic B cells with high expression of BCMA. Interestingly, increased numbers of BCMA tumor cells stimulated anti-BCMA CAR-T cell proliferation in a preclinical model. These anti-BCMA CAR-T cells were also tested in human subjects in a phase I trial, and anti-BCMA CAR-T was successful in all the patients and achieved an overall response rate of 88.2% and sustained tumor remissions up to 12.5 months after therapy. Except for a slight cytokine release syndrome as the most common effect, no other toxicity was observed in the clinical treatment of multiple myeloma.

The one problem of CAR T is its suboptimal efficacy for solid tumors because of lack of specific tumor antigens (100, 101). Development of novel VHH-based CAR T cells against antigens in the tumor microenvironment can circumvent this hurdle. For example, targeting PD-L1, which is widely expressed in the tumor microenvironment, by VHHs-based anti-PD-L1 CAR T cells significantly enhanced immune infiltration and inhibited growth of melanoma and colon adenocarcinoma and improved the survival in mice (94). Another study showed that anti-PD-L1 VHH fused with two cytokines IL2 and IFNγ overcame the delivery barrier caused by an immunosuppressive tumor microenvironment and dense stroma surrounding tumors in an orthotropic pancreatic tumor model (90). Furthermore, the anti-PD-L1 VHH displayed a ~100-fold higher binding affinity than the commercial mAbs 10F.9G2 (90). Therefore, delivery of cytokines by VHHs could be a promising therapy. Another study showed that secretion of anti-CD47 VHHs by CAR T cells can modify the intratumoral immune landscape in immunocompetent animal models (93). CD47 is a tumor antigen that can block phagocytosis of tumor cells by macrophages. Secretion of anti-CD47 VHHs by CAR T cells effectively improved antitumor efficacy in an animal model with an immunocompetent immune system. Further engagement of VHHs in CAR T therapy may support and accelerate personalized, multi-modal immunotherapy based on individual tumor characteristics.

#### 3.2.3 Targeted radionuclide therapy with VHHs

There are five studies included on targeted radionuclide therapy with VHHs (102–106). The radiolabelled VHHs are designed to maximize retention of radioactivity in tumor tissues with overexpression of target antigens and minimize retention of radioactivity in normal tissues. Treatment of VHHs conjugated with radionuclides in in vivo studies resulted in high and specific tumor uptake in multiple myeloma (38), HER2^+^ breast cancer (103, 105), ovarian cancer (103), TS/A mammary carcinoma (102), multiple myeloma (106)and non-Hodgkin lymphoma (104).

One study shows that ^131^I-labelled anti-HER2 VHHs bind to the epitopes that are distinct to the ones recognized by mAbs (Trastuzumab and Pertuzumab) in HER2^+^ breast cancer (103). The VHHs displayed a 4-fold binding efficiency to Trastuzumab-resistant tumor cells compared to Trastuzumab and significantly improve survival of mice, without detected toxicity in an ovarian cancer mice model (103). Another study shows that ^177^Lu-labelled anti-CD20 VHHs is stable in human serum with >91% intact complexes at 144 h post injection (104). The highest tumor uptake of ^177^Lu-labelled anti-CD20 VHHs was at 1.5 h post injection, compared to 120 h for ^177^Lu-labelled anti-CD20 mAbs Rituximab. Furthermore, ^177^Lu-labelled anti-CD20 VHHs significantly improved survival in a lymphoma mice model compared to controls (104). The important drawback of Rituximab is that radioactivity in the circulation is higher than in the tumor at all time points after injection. Also, radioactivity in non-target organs was much higher with Rituximab than with the VHHs.

The heterogeneity of biomarker expression in tumor cells compromises the effectivity of targeted therapy. To develop an efficient targeted radionuclide therapy for a variety of cancers, Bolli et al. proposed to target TAMs, a type of tumor stromal cells, presented in the majority of tumor types that stably express particular targets instead of cancer cells. The radiolabelled monovalent VHHs directed against MMR expressed by TAMs, so called ^177^Lu-labeled anti-α-MMR VHHs, were developed to target stromal cells in a preclinical study (ex vivo and in vivo) (102). This study first optimized the route of administration to avoid undesirable non-tumoral targeting. The co-injection of ^177^Lu-labeled anti-α-MMR VHHs and 100-fold unlabelled bivalent anti-α-MMR VHHs most efficiently reduced non-tumoral uptake of VHHs, while high tumor uptake was maintained. In a terminal animal experiment, 83.3% of mice carrying mammary adenocarcinoma died within 24 days under administration of the monovalent anti-MMR VHHs alone, while all mice survived for longer than 35 days after co-injection with 100-fold unlabelled bivalent α-MMR VHHs. Therefore, blocking non-tumoral binding to decrease therapy-induced toxicity seems to be of high importance in targeted radionuclide therapy. Of note, this therapy targeting MMR in stromal cells pronouncedly outcompeted other common treatments including chemotherapy (doxorubicin, paclitaxel), anti-angiogenic therapy (α-VEGFR2) and immune checkpoint blockade (α-PD1), while the therapeutic efficacy was not improved upon the combination with any of these treatments in the mice model (102). Moreover, ^177^Lu-labeled VHHs successfully targeted stromal cells and delivered a high radiation dose to the stromal cell regions and significantly impaired the progression of tumors that were resistant to conventional therapies (102).

In multiple myeloma, patients are often staged with minimal residual disease (MRD) before cancer relapses. Paraprotein, as an idiotype protein, is expressed on the cell membrane of malignant plasma cells in the MRD stage of multiple myeloma (107). The idiotype involves the presence of a specific set of antigen-binding sites which characterizes an antibody produced by a particular clone (108). The paraprotein sequence is unique to an individual, which makes paraprotein valuable tumor antigens to develop personalized targeted radionuclides. Starting from serum-isolated paraprotein for immunization of llamas, patient-specific anti-idiotype VHHs were successfully generated. These anti-idiotype VHHs (anti-id VHHs) were generated and radiolabelled with ^177^Lu and ^225^Ac for preclinical experiments in a mice model mimicking MRD of multiple myeloma (106). Mice treated with anti-id VHHs had significantly prolonged survival and delayed damage to end-organs compared to the control group. Concluding from this research, radiolabeled anti-id VHHs generate strong therapeutic efficacy against multiple myeloma in the MRD stage, which suggests that radiolabeled anti-id VHHs are valuable therapeutic agents to prevent cancer relapse in multiple myeloma patients.

To overcome limitations due to degradation of radionuclides-labeled VHHs by lysosomes and sustain cellular internalization rate, a recent study radioiodinated anti-HER2 antibodies with ^131^I with the use of a specific radioiodination prosthetic agent (IB-Mal-D-GEEEK containing a D-peptide core) (105). This radioiodinated prosthetic agent was added to facilitate trapping of the radioactivity in the cell after proteolysis of the labeled protein upon internalization in cells (109). The specific tumor-cellular retention of [^131^I]IB-Mal-D-GEEEK-VHHs is lower at 1 hour after administration, but much higher at 24 hours after administration compared to the directly labeled [^125^I]-VHHs (56.9 ± 4.1% at 1h, 58.3 ± 4.6% at 24h versus 61.4 ± 4.3% at 1h and 32.4 ± 2.0% at 24h). Importantly, tumor targeting properties and T/B ratio were also significantly enhanced both in vitro and in vivo for treatment with [^131^I]IB-Mal-D-GEEEK-VHHs compared with directly labelled VHHs ([^125^I]-VHHs). This radiolabeling strategy needs to be evaluated in clinical studies for the treatment of HER2 positive cancers.

#### 3.2.4 VHHs based molecular targeted therapy

Several potentially therapeutic VHHs have been developed to target tumor-specific targets. For instance, in the 26 included studies on molecular targeted therapy (110–135), the targets include the chemokine receptor CXCR7, actin cytoskeleton CapG, receptor tyrosine kinases HER2, EGFR, MET, cluster of differentiation CD7, CD16, CD38, HPV viral-like protein E6, tumor signaling associated proteins BMP2/4, PD-L1, and CD8 T cells.

Earlier studies only focused on the impact of VHHs on tumor cell proliferation, migration and invasion, regardless of specificity, affinity, internalization and biodistribution of the VHHs (110). Anti-EGFR VHHs are the most frequently reported treatment in cancer using VHHs. Anti-EGFR VHHs effectively inhibit proliferation of tumor cells in vitro and outgrowth of solid tumors in vivo by blocking EGF-mediated signaling (122). Other studies often focus on comparative preclinical experiments between novel VHH candidates and conventional mAbs, inhibitors or antagonists. Anti-EGFR VHHs linked to a CPP (non-arginine) resulted in 1000 fold higher cytotoxic activity in tumor cells compared to the small molecular EGFR tyrosine kinase inhibitors in preclinical experiments (126). Viability, mitosis and colony formation of tumor cells were damaged using HER2 targeting VHHs in in vitro experiments (132).

VHH targets are often distinct to epitopes recognized by larger biological molecules, for instance, mAbs, resulting in distinct therapeutic mechanisms. Indeed, Zhai et al. found that VHH enhanced agonistic activity of tumor-necrosis superfamily member 4-1BB by binding a novel epitope of 4-1BB as determined by X-ray crystallography (134). This has the advantage that the VHHs do not affect natural tumor cytotoxicity generated through interaction between 4-1BB and its natural ligand. The most variable portion of immunoglobulin molecules is the third complementarity determining region (CDR3) of the heavy chain (136). In another study, VHHs interacted with EGFR tyrosine kinase residues in the vicinity of their catalytic area (the part to which the natural ligand binds) by using CDR3 and thereby hindered the function of EGFR, impeding tumor cell proliferation and migration (126). A recent study confirmed that VHHs elicit distinct pathways compared to other anticancer drugs, for instance, VHHs blocked the phosphorylation of MET and promoted its degradation via the endo-lysosomal pathway, whereas anti-MET mAbs promoted MET activation (125).

Extended studies further optimized the potential of VHHs for therapeutic efficacy in cancer treatment. Increasing the valence of VHHs from monovalent to multivalent VHHs, for example, trivalent, tetravalent and pentavalent, has been shown to enhance the antitumor effect in a death receptor 5-targeting preclinical study (116). Producing tetravalent or pentavalent VHHs highly increased the binding affinity compared to monovalent VHHs. In addition, tetravalent and pentavalent VHHs both maximally induced activation of caspase-8 and caspase-3/7, resulting in the formation of death-inducing signaling complexes, leading to significant enhancement in anticancer response in vitro and in vivo. In general, the valence of VHHs is one of the key factors that determines the anticancer capability of VHHs as anticancer drugs. Studies suggested that the binding affinity and targeting potency of VHHs vary with their valence (68, 121). However, one study found that there is no significant difference between monovalent and bivalent VHHs in respect to their tumor targeting potential (112). The discrepancy between the different studies is possibly due to a limited amount of epitopes being expressed on cancer cells, which limits the measurable effect on affinity by increasing agent concentrations. Otherwise, the latter study supports the existence of an affinity threshold value beyond which no further improvement of tumor binding can be achieved. Furthermore, the study also found that higher affinity does not necessarily enhance tumor penetration. This is probably due to the presence of a binding site barrier effect, resulting from VHHs binding to the first epitopes encountered at the tumor cells in the superficial tumor layer, which impedes deeper penetration of the VHHs to reach the core of the tumor (137). One study reported that the mixture of four VHHs targeting four distinct epitopes of HER2 improved target binding potential and therapeutic efficacy in an in vitro model (114). This suggests that targeting different epitopes of the same antigen simultaneously might be a potential strategy to improve efficacy.

Our group developed two VHHs C4C4 and C8C8 directed against BMP4 and BMP2/4, respectively. We demonstrated that specificity, effectiveness and affinity of C4C4 and C8C8 are higher than conventional anti-BMP2 and/or BMP4 antibodies (138, 139). We also found that bivalent VHHs C4C4 and C8C8 lead to higher affinity than monovalent C4 and C8, respectively (139). Furthermore, in vitro experiments confirmed that C4C4 and C8C8 inhibit cell migration and chemo-resistance to cisplatin in primary esophageal adenocarcinoma (EAC) cell line. In a patient-derived xenograft model, IRDye800cw-labelled C4C4 and C8C8 effectively target BMP4 and BMP2/4, respectively, and inhibit tumor growth (119). Overall, our study demonstrated that VHHs inhibited tumor growth and aggressiveness of EAC in vitro and in vivo through inhibiting BMP4 and BMP2/4 (119). Furthermore, C4C4 and C8C8 also were able to inhibit the growth of esophageal adenocarcinoma premalignant lesions, known as Barrett`s esophagus in an in vivo organoid model (111).

Although VHHs contain no Fc region, a study generated anti-CD38 VHH-Fc by genetically fusing VHH with the Fc-domain of human IgG1 (123). The VHH-Fc with a high specificity and affinity binds to a different epitope than the conventional mAb Daratumumab. The VHH-Fc induced potent antibody-dependent cellular cytotoxicity in a myeloma cell line and primary multiple myeloma cells. Furthermore, the VHH-Fc inhibited tumor growth and prolong survival time compared to a control and Daratumumab group in a mouse xenograft model.

VHHs that simultaneously target immune cells, such as NK cells in the tumor microenvironment, next to tumor cells, result in a more aggressive antitumor therapy than treatment of targeting tumor cells only. Indeed, innovative studies have shown that bispecific and even tri-specific VHHs which interact with NK cells elicited robust cytotoxicity and cytolysis of tumor cells due to stronger activity of the induced NK cell responses (128, 129). The tri-specific VHHs trigger significant NK cell expansion and further enhance immunity response of NK cells on tumor cells as shown in in vivo experiments. The advantage of these multi-specific VHHs lies in the combination of molecular targeted therapy on tumor cells and immunotherapy focused on NK cells and the removal of non-specific killing by NK cells. In another application in immune therapy, anti-PD-L1 VHH in combination with an anti-OX40 antibody were used to construct the bispecific antibody PD-L1/OX40, that had better antitumor effects than the anti-PD-L1 VHH alone, anti-OX40 antibody alone, or a combination therapy of anti-PD-L1 VHH plus anti-OX40 in colon cancer and lung cancer mice models (118).

To address tumor cell resistance in conventional therapies, a VHH was designed as an immunotoxin by fusion of the VHH with peptide toxins (de-immunized pseudomonas exotoxin) with distinctive anticancer mechanisms (115, 127). The novel VHH-based immunotoxin displayed superior antigen-restricted cytotoxicity against tumor cells and evident survival benefit in a mice model (127). Further optimization of the immunotoxin was performed by addition of a streptococcal albumin binding domain and the removal of T-cell epitopes, leading to longer serum half-life and lower immunogenicity (115). It is important to note that adding albumin to immunotoxins may lower the effective dose, as shown in mouse liver cancer xenografts (115). Another study demonstrated the targeting specificity of humanized anti-CD7 immunotoxin in vitro and in vivo (133). The anti-CD7 immunotoxin significantly inhibits the proliferation of T-cell acute lymphoblastic leukemia cells and extends the survival time of mice in a tumor transplant model and patient tumor-derived xenograft model by exerting a cytotoxicity function via endocytosis into the cytoplasm of CD7-positive cells. Moreover, study reported that anti-MHC-II VHH-drug conjugates are superior than commercial mAbs on pharmacokinetics, such as internalization and circulatory clearance and display efficient anticancer outcomes for an aggressive murine B-cell lymphoma (11).

VHHs are also used for targeting intracellular protein, besides proteins on the cell-membrane. In a breast cancer study, breast cancer cells expressing anti-CapG VHHs lost the capability of migration and invasion (130). Tumor-bearing mice inoculated with breast cancer cells expressing anti-CapG VHHs showed significant slower tumor growth and prevention of lung metastasis compared to the control group. Other studies were performed to enhance tumor cell penetration and to increase the intracellular concentrations of VHHs. Solid tumors are characterized by high interstitial fluid pressure, hypoxia and homeostatic imbalance, contributing to the difficult penetration of anticancer drugs within tumor tissues. The anticancer activity of anti-EGFR VHHs was further enhanced by introduction of a CPP called iRGD in gastric cancer therapy in ex vivo and *in vivo* models (124). Anti-EGFR VHHs fused with iRGD significantly improved penetration and were able to reach the core area of the tumor mass in established multicellular spheroids. The favorable penetration ability of anti-EGFR-iRGD VHHs was reflected by the change of tumor volume in a mice model. The tumor volume shrunk by about 63.7% for anti-EGFR-iRGD VHHs, outcompeting the anti-EGFR VHHs group. Combining other anti-cancer drugs including DOX (20 µg/mL), liposomes (1.5 µg/mL) and mAbs Bevacizumab (1 mg/mL) with anti-EGFR-iRGD VHHs significantly enhanced their penetration capability and tumor inhibiting activity in mice models (124). Moreover, considering that leaky vasculature exists in most solid tumors, a delivery carrier so called PEGylated liposomes were investigated in the therapy of HER2 overexpressed cancer in vitro (114). This approach displayed great therapeutic potential by combining the drug delivery advantages of liposomes and tumor-specific binding capabilities of the VHHs.

One recent study has extended the utilization of VHHs in cancer treatment. VHHs as delivery carriers have been used for development of cancer vaccines against melanoma. These therapies significantly decreased tumor growth in a mice model (113). Woodham and colleagues established a therapeutic vaccine platform based on VHHs for treatment of HPV induced cancer (131). The novel vaccine, VHH_CD11b_-E7, is composed of a HPV tumor antigen peptide E7 and a VHH directed against CD11b as expressed on antigen-presenting cells by site-specific conjugation. The VHH-conjugated vaccine could elicit potent cellular immunity to attack tumor cells in established HPV positive tumors, as opposed to regular prophylactic vaccinations against HPV. The VHH_CD11b_-E7 recognized an epitope, which is different from the epitope recognized by the conventional anti-CD11b mAbs. This could have contributed to the high affinity of VHH_CD11b_-E7 for CD11b expressing cells. The extent of CD8^+^ T-cell response determines the efficacy of the therapeutic vaccine against HPV induced cancers (140). An enhanced cellular immune response could be attributed to VHH_CD11b_-E7, as suggested by a two-fold increase in the number of CD8^+^ T cells compared to E7 alone. VHH_CD11b_-E7 also significantly outperformed E7 alone in terms of tumor regression. Although VHH_CD11b_-E7 is developed as a therapeutic vaccine, prophylactic vaccination with VHH_CD11b_-E7 also significantly lowered the tumor burden and improved overall survival compared to E7 alone in mice. Overall, the application of tumor antigens conjugated with VHHs, which are of importance to acquire an immune response, such as VHH_CD11b_-E7, should be considered in the clinic as a potential therapeutic vaccine for HPV induced cancers.

In addition to their use in vaccines in HPV induced cancers, VHHs can also be designed to directly target oncoproteins such as oncoproteins E6 and E7 which play key roles in HPV16 induced cancer (120, 135). This showed effective inhibition of tumor growth, which suggests that targeting E6/E7 in therapy of HPV16 induced cancer holds great promise.

Therapeutic stem cells can be designed to secrete VHHs in the treatment of Basal-like breast cancer (BLBC). BLBC is associated with poor prognosis and survival with a high incidence of brain metastasis and poor delivery of drugs through the BBB. In the past decade, intrathecal stem cell therapy has been investigated and applied in patients with neurodegenerative diseases and trauma (141–143). Building on these experiences, scientists developed human mesenchymal stem cells (hMSCs) secreting VHHs (E_V_DR_L_) directed against EGFR and DR_L_ simultaneously (117). hMSCs-E_V_DR_L_ treatment significantly lowered tumor volumes and prolonged survival in mice BLBC models of brain metastasis. In a mice model, histopathology showed hMSCs-E_V_DR_L_ being widely distributed at residual tumor cells along with the brain vasculature after surgical resection of the primary tumor, suggesting a perivascular niche of micro-metastasis. Treatment with hMSCs-E_V_DR_L_ significantly increased macro-metastasis-free survival and overall survival of mice. In a mice model of BLBC leptomeningeal metastasis, hMSCs-E_V_DR_L_ survived in cerebrospinal fluid (CSF) space longer than 2 weeks after intrathecal injection. This suggests that hMSCs-E_V_DR_L_ can overcome the therapeutic difficulty of fast clearing drugs by CSF. Treatment with hMSCs-E_V_DR_L_ exhibits powerful therapeutic efficacy by targeting residual invasive tumor cells after surgical resection in the perivascular niche and leptomeningeal metastasis of BLBC. Therefore, the delivery of stem cell secreting VHHs could be an effective therapeutic strategy for BLBC treatment.

## 4 Discussion

### 4.1 VHHs instead of conventional mAbs

VHHs are emerging as innovative imaging and therapeutic tools, which can be considered as promising alternatives for the commonly used conventional mAbs. In several preclinical studies, their superiority in terms of target specificity, affinity and anticancer effects has been demonstrated, while clinical applications are warranted. Multiple preclinical and clinical studies in cancer imaging showed that VHHs can circumvent many of the unavoidable drawbacks caused by mAbs’, this includes delayed imaging times, high radiation burden and poor T/B ratios (57, 144).

### 4.2 Easy and cheap to make VHHs

Cheap expression and easily conjugation with a variety of agents make VHHs versatile imaging and therapeutic tools (90). VHHs can be used for site-specific conjugation with payloads such as radioisotopes, fluorophores, photosensitizers, drugs and cytokines.

### 4.3 High specificity and affinity

High specificity and affinity are two most frequently demonstrated advantages in the included studies for VHHs in cancer imaging and therapy. Imaging with VHHs not only can avoid false-negative diagnostic results due to heterogenetic expression of target antigens, but also more clearly visualize and quantitatively detect the heterogenetic status of target protein expression in different primary and metastatic lesions.

### 4.4 Less toxicity

Rapid circulation clearance and high specificity confine VHHs to tumor sites, leading to less potential systemic toxicity compared to mAbs. Besides kidney retention, no symptoms of toxicity have been found in the preclinical studies and the only two existing clinical studies (23, 92).

### 4.5 Easy administration

We found that the time interval between administration and performance of imaging or treatment is much shorter for VHHs compared to mAbs. The superior features of VHHs lead to fast accumulation in specific tumor sites and rapid clearance from circulation and higher T/B ratio. This way, imaging and treatment can be performed very shortly after administration, which would likely reduce the burden for patients and bring benefits in terms of flexibility, operational costs and hospital management. With regard to photodynamic therapies, the small size of VHHs are beneficial for the efficacy of PDT. The half-life of the reactive oxygen species generated by PS are quite short (<40 ns), leading to short travel distances (<20 nm) (79). Therefore, reactive oxygen species released by VHH-PS close to cellular membrane or cellular organelles may be in favor of potency.

### 4.6 Treatment strategy

We found that the majority of VHHs recognize distinct epitopes than respective mAbs (28, 66, 103, 122, 131, 134). Therefore, imaging with VHHs can also be used as companion diagnostics for real-time monitoring expression changes of targeted antigens during targeted therapy with mAbs. Furthermore, VHHs could provide complemental therapy when directed against the same target as existing mAbs therapy. VHHs also can be used as single agents or combined with conventional systemic anticancer therapies (119). Drug resistance is common in mAbs therapy because the epitopes recognized by the mAbs can mutate and resist binding. The VHHs that recognize alternative epitopes could provide alternative therapy or add-on therapy for mAbs-resistant cancer (26).

### 4.7 Limitations of the review

Lacking quantitative analysis is a limitation of this review, due to inter-study heterogeneity. Most studies are preclinical studies, investigating different types of cancers. The outcomes of the different studies are difficult to compare, because of the use of different cell lines for the same type of cancer, including several primary cell lines and various commercial cell lines. The studies investigated many different VHH conjugates, with VHH conjugated to various radionuclides in radiolabelling imaging and targeted radionuclide therapy and different types of fluorescent groups for optical imaging. Moreover, the VHHs in these studies were designed to target distinct antigens. We also found that the comparators are different in the studies. Some studies compared the VHH group to the respective mAbs group, whereas other studies compared VHH groups to other VHH groups. Commercial inhibitors and chemotherapy are also in the control groups as comparators. Outcomes in the studies also vary. Some studies focus on T/B ratio, cellular internalization, biodistribution in target organ and non-target organs, whereas some other studies focus on blood clearance time and tumor uptake. Overall, the heterogeneity in terms of outcomes between the studies makes it difficult to perform a systematic review (meta-analysis).

The majority of the included studies employed in vivo experiments in the study design, which is important for the investigation of VHHs. The superior features of VHHs arise from their small size and high target specificity, which can be displayed only in in vivo experiments in terms of good tissue penetration, rapid circulation clearance and high T/B ratio. However, several studies only performed in vitro experiments.

From the 73 included studies, we found that nearly all studies hold promise to be beneficial for clinical application. For several studies, it seems that the currently available technology is limited and a lot of additional research has to be performed before results can be translated to clinic. For instance, two studies developed anti-HPV E6 and anti-HPV E7 VHHs and confirmed their anti-cancer effects in vitro and/or in vivo (120, 135). E6 and E7 are intracellular proteins and therefore the studies adopted a strategy to have cancer cells express VHHs. Although the studies showed attractive results, with the current level of technology it is not possible to inhibit tumors by making patients’ cancer cells express VHHs. Likewise, another study involving breast cancer cells expressing anti-CapG VHHs also raises the same concerns (130).

### 4.8 Conclusion

We discussed various preclinical studies focusing on the suitability of VHHs for imaging and therapy of different types of cancer. This review shows that based on the current literature VHHs are superior in terms of affinity, specificity, internalization, immunogenicity, non-targeted cytotoxicity, efficacy and safety compared to conventional mAb and its derivatives. The only two clinical studies with human subjects on VHHs showed that the application of VHHs in imaging for breast cancer, and CAR-T cell therapy for multiple myeloma were successful in all patients with safe and effective results (23, 92). It seems that VHHs hold great promise for imaging and therapy of cancer. However, despite the numerous potential in applications of VHHs in molecular targeted imaging and therapy, more clinical studies with important data regarding toxicity and efficacy in human subjects are needed. These studies are urgently-needed to enable clinical translation and implementation with the potential of improving patient outcomes. Overall, VHHs could be attractive novel targeted therapeutics, given the fact that VHHs already exemplified promising translational potential in preclinical studies.

## Author contributions

SL: Conceptualization, Funding acquisition, Investigation, Methodology, Writing – original draft, Writing – review & editing. SH: Writing – review & editing. KK: Conceptualization, Funding acquisition, Methodology, Supervision, Writing – review & editing.
